# The CONSORT Patient-Reported Outcome (PRO) extension: implications for clinical trials and practice

**DOI:** 10.1186/1477-7525-11-184

**Published:** 2013-10-29

**Authors:** Melanie Calvert, Michael Brundage, Paul B Jacobsen, Holger J Schünemann, Fabio Efficace

**Affiliations:** 1 MRC Midland Hub for Trials Methodology Research, School of Health and Population Science, University of Birmingham, Birmingham, UK; 2 Department of Medical Oncology, Queen’s University, Kingston, ON, Canada; 3 Department of Health Outcomes and Behavior, Moffitt Cancer Center and Research Institute, Tampa, Florida, USA; 4 Departments of Clinical Epidemiology & Biostatistics and of Medicine, McMaster University Health Sciences Centre, Ontario, Canada; 5 Head, Health Outcomes Research Unit, Italian Group for Adult Hematologic Diseases (GIMEMA), GIMEMA Data Center, Via Benevento, 6, 00161 Rome, Italy

**Keywords:** Quality of life, CONSORT PRO, Reporting, Clinical trials

## Abstract

To inform clinical guidelines and patient care we need high quality evidence on the relative benefits and harms of intervention. Patient reported outcome (PRO) data from clinical trials can “empower patients to make decisions based on their values” and “level the playing field between physician and patient”. While clinicians have a good understanding of the concept of health-related quality of life and other PROs, evidence suggests that many do not feel comfortable in using the data from trials to inform discussions with patients and clinical practice. This may in part reflect concerns over the integrity of the data and difficulties in interpreting the results arising from poor reporting.

The new CONSORT PRO extension aims to improve the reporting of PROs in trials to facilitate the use of results to inform clinical practice and health policy. While the CONSORT PRO extension is an important first step in the process, we need broader engagement with the guidance to facilitate optimal reporting and maximize use of PRO data in a clinical setting. Endorsement by journal editors, authors and peer reviewers are crucial steps. Improved design, implementation and transparent reporting of PROs in clinical trials are necessary to provide high quality evidence to inform evidence synthesis and clinical practice guidelines.

## Background

**Figure 1:**
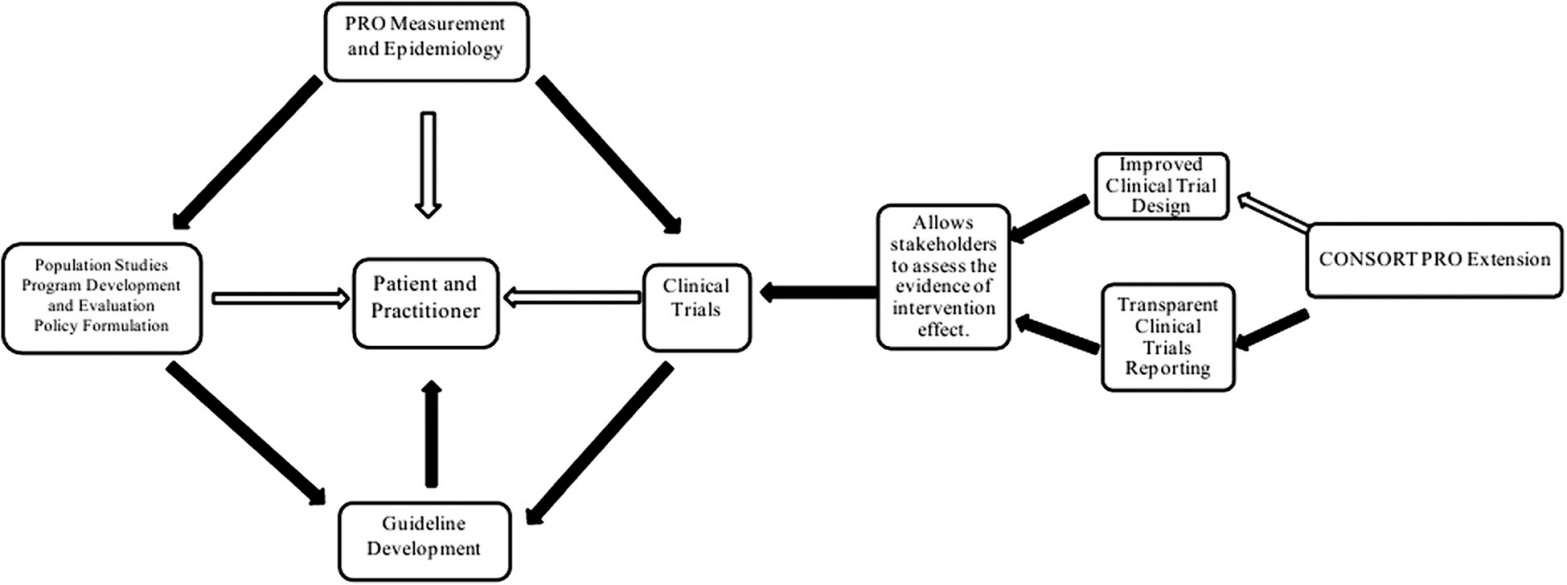
**Model for the use of PRO data to inform patient care.** Major routes are indicated by solid arrows whilst other possible routes are indicated by unfilled arrows. The potential impact of the CONSORT PRO Extension on evidence from clinical trials and links to guideline development are indicated. Adapted from Till et al. [3].

Randomized controlled clinical trials (RCTs) can provide high-quality data regarding the impact of study interventions on patient outcomes, and remain the ‘gold standard’ of evidence regarding both the benefits and harms associated with an intervention. Over the last twenty years, the number of clinical trials that assess patient reported outcomes (PROs) has substantially increased [1]. PROs can be defined as a “measurement of any aspect of a patient’s health status that comes directly from the patient (i.e. without the interpretation of the patient’s responses by a physician or anyone else” [2] and include health-related quality of life (HRQL), symptoms, satisfaction and adherence to medication. These subjective measures of outcome help evaluate the burden of disease and treatment from the patients’ perspective. In the conceptual framework developed by Till and colleagues adapted in (Figure 1) [3], PRO data from clinical trials may directly inform patients and practitioners, or may indirectly inform clinical practice through evidence synthesis into clinical practice guidelines.

The quality of data, including PROs, from trials may be threatened by the trial design, execution, and reporting. Like any other outcome being considered, PROs should be pre-specified, relevant and appropriate measure(s) [4]. Failure to address a number of specific issues at the time of protocol writing or in the final trial report, such as the PRO hypothesis, timing and mode of assessment and strategies to manage missing data, can undermine the clinical validity of findings and limit translation of these into clinical practice [5].

All RCT outcomes, including PROs, should be completely and transparently reported. Anything less may diminish the usability of the trial results by clinicians and is an inefficient use of resources. High-quality reporting of PROs in clinical trials is required to allow readers to assess the validity of the PRO findings and assess any potential sources of bias. Evidence suggests, however, that reporting of PROs remains sub-optimal across RCTs [6,7]. Previous studies estimating the proportion of PRO-RCTs that were likely to robustly inform clinical decision-makings in prostate cancer, for example, have found that only one third of PRO-RCTs published between 1980 and 2001 did so [8].

In a review of 794 RCTs published up to 2008 across a range of clinical settings, only 56% of articles provided a rationale for the selected PRO, 50% provided a PRO hypothesis, 28% provided information about PRO missing data and 64% discussed the PRO findings in the context of the other trial outcomes [7]. Although the PROs of some RCTs were reported in an expanded supplementary publication, this only occurred for 15% of trials at the time of review, and the detailed PRO publications often appeared in methodologically focused journals. In cancer RCTs methodological drawbacks have often hampered a critical appraisal of results [9,10].

Additional qualitative evidence suggests that these patterns of PRO reporting negatively impact on the effective use and dissemination of RCT PRO findings to clinical practice. A study of 30 academic oncologists examining their attitudes and behaviors regarding HRQL data from clinical trials indicated that, while these data were typically valued by clinicians and felt to benefit their patients, problems with the manner in which these data were reported in the literature limited their use in clinical practice [5]. The data were considered to be less accessible than outcomes such as survival or the adverse effects of treatment. Many clinicians felt that HRQL/PRO data were frequently reported in a manner that is difficult to interpret and integrate with clinical outcomes, and were often reported months or years after the primary trial publication. Clinicians also wanted to be assured that the appropriate measurement instrument was used, that the results were not biased by missing data, that results were not “cherry picked” (i.e., chance findings arising from multiple testing), and that reported changes in PROs were clinically meaningful.

In this paper, we focus on how the recently developed CONSORT (Consolidated Standards of Reporting Trials) PRO extension [11] may be implemented to inform individual patient care and clinical guidelines as illustrated in (Figure 1).

### The CONSORT PRO extension as a vehicle for change and challenges to its implementation

The CONSORT statement aims to encourage transparent and complete reporting of clinical trials and is associated with improved reporting of trials [12]. Importantly, the CONSORT group has a proven ability to reach consensus on reporting guidelines and to promote their successful implementation. Extensions to the original CONSORT statement have provided guidance for specific trial designs, interventions, and specific outcomes but until recently did not provide specific guidance for PROs [13]. Such guidance was clearly required since multiple factors threaten the quality of PRO reporting: researchers must design their RCTs with appropriate PRO implementation and execution, and authors must find a balance between providing sufficient detail to adequately report PRO results within the context of other study outcomes while still adhering to journal requirements for manuscript length and structure. Reviewers and editors must find the optimal balance between demanding that certain reporting standards be included in the context of the same journal constraints.

The development of a CONSORT PRO extension was thus undertaken, led by a Task Force of the International Society for Quality of Life Research (ISOQOL) and guided by the methodological framework proposed by the EQUATOR Network [14]. Briefly, a systematic search was used to identify candidate reporting standards for PROs [15]. An electronic survey of stakeholder groups was used to obtain experts’ judgments of the importance of each candidate standard for PRO reporting and the survey results were then debated by a face-to-face meeting of stakeholders to achieve consensus on items that should comprise a CONSORT extension. The meeting agenda included: a review of relevant evidence, discussion of the rationale for including or excluding items in a PRO reporting standards checklist from the point of view of the multiple stakeholders at the table, anonymous voting on the proposed standards, the development of a draft CONSORT extension, and the development of a knowledge translation and dissemination strategy [13,14].

The group was ultimately able to reach consensus on five “extension” statements to the 2010 CONSORT guidance that each address a key reporting item considered critical for quality reporting from all types of RCTs where PROs are a primary or “important” secondary outcome as shown in Table 1. Important secondary outcomes refer to pre-specified domains that are focus of the principal PRO analyses [11]. In addition, critical components of the existing 2010 CONSORT guidance that were critically relevant to PRO reporting were expanded by “elaboration” statements. The rationale for each of the 5 items comprising the CONSORT PRO extension are briefly summarized in Table 1. Readers are referred to the CONSORT PRO Extension for further information on the rationale for each item, examples of reporting and elaboration of the existing CONSORT 2010 statement in relation to PROs [11]. These guidance statements, if implemented by authors and journal reviewers/editors, could potentially improve reporting and facilitate interpretation of PRO results for use in clinical practice.

**Table 1:** Summary of the CONSORT PRO extension items

**CONSORT 2010 statement**	**PRO extension**	**Brief rationale for extension**
Structured summary of trial design, methods, results, and conclusions	The PRO should be identified in the abstract as a primary or secondary outcome	Explicitly identifying PROs in the RCT abstract will facilitate indexing and identification of studies to inform clinical care and evidence synthesis.
Specific objectives or hypotheses	The PRO hypothesis should be stated and relevant domains identified, if applicable.	PRO measures may be multi-dimensional and may assess patient status at several time points during a RCT. A pre-specified hypothesis reduces the risk of multiple statistical testing and selective reporting of PROs based on statistically significant results.
Completely defined pre-specified primary and secondary outcome measures, including how and when they were assessed.	Evidence of PRO Instrument validity and reliability should be provided or cited if available including the person completing the PRO and methods of data collection (paper telephone electronic other).	This information will allow readers to assess the validity, reliability and appropriateness of the PRO being used.
Statistical Methods used to compare groups for primary and secondary outcomes	Statistical approaches for dealing with missing data are explicitly stated.	Missing PRO data is a potential source of bias. A number of methods for dealing with missing data are available with different strengths and limitations which should be described to facilitate interpretation.
Trial limitations addressing sources of potential bias, imprecision, and, if relevant multiplicity of analyses	PRO-specific limitations and implications for generalizability and clinical practice should be discussed.	PRO specific limitations may influence the generalizability of results and use in clinical practice.
Generalizability (external validity, applicability) of trial findings		

Authors are referred to the CONSORT PRO Extension paper for further explanation and the full list of items [11]. The CONSORT PRO extension should be used in conjunction with the CONSORT checklist and relevant extensions as appropriate for the study design.

Experience with the CONSORT Statement suggests that efforts to implement the CONSORT PRO Extension will face a number of challenges. First and foremost is obtaining journal endorsement for the CONSORT PRO Extension. A 2008 analysis of “Instructions to Authors” for 165 high impact factor medical journals found that only 38% mentioned the main CONSORT statement in their online instructions [16]. Moreover, of the four CONSORT extensions that were examined, no single extension was mentioned on more than 3% of journal websites. Evidence suggests that reporting is improved in those journals with a policy of active implementation of CONSORT [17].

Theory and research on dissemination and implementation in health care settings suggests ways to promote greater adoption of the CONSORT statement and its extensions [18]. Success can be enhanced when efforts extend beyond typical “push” approaches (i.e., publishing and publicizing new information), to include “pull” approaches (i.e., creating demand for change among the target audience). One possible way to create demand for endorsement is to have regular public reporting audit of those journals that do and do not mention CONSORT or its extensions in their online Instructions to Authors.

Even when CONSORT is endorsed there is considerable variation in how it is implemented at journals. Without strong direct language, for example, in a journal’s Instructions to Authors section, prospective authors will not have clear guidance about how they need to comply with journal endorsement. Plans to develop online resources which provide specific examples taken from publications should facilitate adherence to the new CONSORT-PRO checklist. Problems may be encountered; however, with consistency in the operationalization of “important secondary outcomes” since this term is not currently part of the standard clinical trial lexicon. In addition, problems may be encountered with how authors interpret the requirement that evidence of PRO instrument reliability or validity be provided or cited if available. Several questions arise in this regard. Is it sufficient to cite the original article that describes an instrument’s psychometric properties? Ideally further information on reliability should be reported in relation to the population of study in the clinical trial. Should supporting evidence also be provided for an instrument’s responsiveness to change? Should additional psychometric information be provided for translations of instruments? In addressing these issues, authors will need to stay abreast of evolving standards for PROs and their use in clinical research [2,19].

### Potential impact of the CONSORT PRO extension on the quality of clinical trial reporting

The need for improvement in the reporting of PROs is one of the principal reasons for developing the CONSORT PRO Extension. How likely is it, however, that publication and implementation of the CONSORT PRO Extension will have the desired positive impact? A recent systematic review and meta-analysis provides some insight. The review encompassed published studies evaluating the completeness of reporting of randomized controlled trials in medical journals based on CONSORT Statement criteria [12]. Data for the review were drawn from 53 publications encompassing over 16,000 randomized clinical trials. Findings showed that 25 of 27 comparisons of the completeness of reporting favored CONSORT-endorsing journals over non-endorsers, with five of these comparisons yielding statistically significant differences. These findings strongly suggest that endorsement of the CONSORT PRO Extension will over time result in improvements in the reporting of PROs in clinical trials.

### A Real-World Example from Oncology: evidence from prostate cancer RCTs evaluated against the CONSORT PRO extension items

In an effort to provide current evidence on the level of reporting according to the new CONSORT PRO extension items, in Table 2 we provide the proportion of recently conducted PRO-RCTs in prostate cancers that have addressed such issues. A systematic literature search, identifying all RCTs published with a PRO component (published between 2004 and 2012) in prostate cancers found 65 studies [20]. All these RCTs were scrutinized based on a number of methodological criteria, including the CONSORT PRO extension items and data are summarized in Table 2. A remarkable finding is that only 18% of studies documented statistical methods for addressing PRO missing data. Also, only 23% reported methods for PRO administration. These very low percentages, underscore that some specific items reported in the new CONSORT PRO extension, are in urgent need of improvement to raise confidence in the solidity of PRO data stemming from these RCTs.

**Table 2:** Level of reporting in recently conducted prostate cancer RCTs (65 studies published between 2004 and 2012) according to the new CONOSRT PRO extension

**Consort 2010 statement**	**PRO extension**	**Level of reporting, n (%)**
Structured summary of trial design, methods, results, and conclusions	The PRO should be identified in the abstract as a primary or secondary outcome	59/65 (91%)
Specific objectives or hypotheses	The PRO hypothesis should be stated and relevant domains identified, if applicable.	24/65 (37%)
Completely defined pre-specified primary and secondary outcome measures, including how and when they were assessed.	Evidence of PRO Instrument validity and reliability should be provided or cited if available including the person completing the PRO and methods of data collection (paper telephone electronic other).	43/65 (66%), Yes, for all PRO instruments used in the study.
		15/65 (23%), reported “mode of administration”
Statistical Methods used to compare groups for primary and secondary outcomes	Statistical approaches for dealing with missing data are explicitly stated.	12/65 (18%)
Trial limitations addressing sources of potential bias, imprecision, and, if relevant multiplicity of analyses	PRO-specific limitations and implications for generalizability and clinical practice should be discussed.	23/65 (35%) PRO-specific limitations discussed.
		37/65 (57%) Implications for generalizability
Generalizability (external validity, applicability) of trial findings		

Data extracted from Efficace et al. [20].

### Implications of the CONSORT PRO extension for RCT design

The CONSORT PRO extension has important implications for trial design, although design decisions may go beyond simply what should be reported. The problem of PRO missing data illustrates this distinction. Difficulties with PRO data collection and compliance have historically been considered the major barriers to the successful implementation of PRO in clinical trials [21]. The CONSORT PRO extension states that the amount of missing data and way that this has been addressed in the analysis should be reported. The analysis plan should clearly be pre-specified at the design stage, but in addition methods to prevent or minimize missing data in the trial should be stated. In addition the CONSORT PRO Extension stipulates that evidence of PRO instrument validity and reliability be included as part of the manuscript submission. This criterion has the potential to foster greater consideration of a PRO measure’s psychometric properties at the time of protocol design, thereby promoting greater use of measures with demonstrated reliability and validity. Likewise, consider the CONSORT PRO Extension item stipulating that the PRO hypothesis be stated in the manuscript, with relevant domains identified if applicable. This criterion has the potential to foster greater pre-specification of PRO hypotheses at the time of protocol development, thereby reducing the likelihood of selective reporting of PRO results in manuscript. It should be noted, however, that specification of outcomes in a protocol may not be sufficient to eliminate the problem of selective result reporting. Indeed, an audit comparing protocols submitted to a leading medical journal and the published reports based on those protocols found numerous discrepancies between the pre-specified outcomes and the reported outcomes [22]. Additional efforts, such as providing manuscript reviewers with access to the original protocols, would appear to be necessary to meaningfully address the issue of selective reporting.

Within this framework, it is possible to envisage a positive impact of endorsement of the CONSORT PRO Extension not only in improving to the quality of clinical trial reporting but also on PRO trial design. The quality of certain aspects of clinical trials incorporating PROs are likely to improve as a direct result of widespread adoption of the CONSORT PRO Extension, while others will require more multi-faceted efforts on the part of the research community.

### Improved PRO trial design and reporting: implications for guideline development

Individual randomized trials may either by chance or as a result of bias not represent the best possible estimates of an effect of an intervention on PROs. Thus, single small RCTs are rarely informative for evidence based health care decisions. Evidence-based health care decisions require systematic reviews to more completely consider the possible influence of bias and chance on health care decisions.

Systematic reviews of intervention effects alone, however, are also insufficient for evidence-based health care decision-making. In addition to information about effects of interventions, health care decisions require consideration of issues around the clinical state and circumstances including baseline risks for a certain outcome, the populations and societal values and preferences, estimates of effect and expertise to bring all of that together. Practice guidelines are recommendations that are intended to assist providers, recipients of health care and other stakeholders to make informed decisions and can take on the role of adequate support tools [23].

PROs can inform evidence-based recommendations in a number of ways. PROs address what matters to patients and their use in clinical trials can provide the information that is critical for decision making in practice guidelines. PROs also can provide information about the importance of other outcomes, non PROs, by conducting explicit value and preference elicitation exercises. Transparent communication of both the confidence in an estimate of effect on a PRO, which is also called the quality of the evidence [24,25], and the estimate itself remains challenging. The GRADE Working Group has developed a system to assess the confidence and the likelihood of an estimate of effect on PRO that facilitates communication, which may be incorporated formally in the review process. The group is now focusing on a structured approach to assessing the confidence in estimates of values and preferences that influence the development of health care recommendations. The system is used by over 70 organizations worldwide. Using such systems has become a requirement in most standards on clinical practice guideline development [26,27].

## Conclusion

In conclusion, robust methodology and accurate reporting of data are crucial when evaluating PROs in clinical trials in order to provide health care providers and policy-makers with a transparent message about the impact of a given drug or a novel therapeutic approach on the patient’s health status. Scientific publications stemming from a poor study design or simply reporting insufficient information can mislead readers when interpreting study outcomes. Such publications are wasteful, both in terms of opportunities for providing better evidence, research funding, the effort of everyone involved from concept to paper – including but not confined to investigators, ethics committees, patients, trial co-ordinators, statisticians, journal staff, reviewers and finally readers. The new CONSORT PRO extension aims to raise quality standards and help bridge the gap between PRO-trial based outcomes and clinical practice by increasing clinician confidence in published PRO evidence.

## Competing interests

MC, MB, PJ and FE all contributed to the development of the CONSORT PRO Extension and publication.

## Authors’ contributions

All authors contributed to drafts of the manuscript, read and approved the final version for publication.
